# Synthesis of Highly Conductive Poly(3-hexylthiophene) by Chemical Oxidative Polymerization Using Surfactant Templates

**DOI:** 10.3390/polym14183860

**Published:** 2022-09-15

**Authors:** Sanhanut Kesornsit, Chatrawee Direksilp, Katesara Phasuksom, Natlita Thummarungsan, Phimchanok Sakunpongpitiporn, Kornkanok Rotjanasuworapong, Anuvat Sirivat, Sumonman Niamlang

**Affiliations:** 1 Conductive and Electroactive Polymers Research Unit, The Petroleum and Petrochemical College, Chulalongkorn University, Bangkok 10330, Thailand; 2 Center of Excellence on Petrochemical and Materials Technology (PETROMAT), Chulalongkorn University Research Building, Soi Chula 12, Phayathai Road, Bangkok 10330, Thailand; 3 Department of Materials and Metallurgical Engineering, Faculty of Engineering, Rajamangala University of Technology Thanyaburi, Pathumthani 12110, Thailand

**Keywords:** poly(3-hexylthiophene), emulsion polymerization, electrical conductivity, conductive polymer

## Abstract

Poly(3-hexylthiophene) (P3HT) was systematically synthesized by chemical oxidative polymerization in chloroform with ferric chloride (FeCl_3_) as the oxidizing agent and various surfactants of the shape templates. The effects of 3HT: FeCl_3_ mole ratios, polymerization times, and surfactant types and concentrations on the electrical conductivity, particle shape and size were systematically investigated. Furthermore, dodecylbenzenesulfonic acid (DBSA), p-toluenesulfonic acid (PTSA), sodium dodecyl sulfate (SDS), and sodium dioctyl sulfosuccinate (AOT) were utilized as the surfactant templates. The P3HT synthesized with DBSA at 6 CMC, where CMC stands for the Critical Micelle Concentration of surfactant, provided a higher electrical conductivity than those with PTSA, SDS and AOT. The highest electrical conductivity of P3HT using DBSA was 16.21 ± 1.55 S cm^−1^ in which the P3HT particle shape was spherical with an average size of 1530 ± 227 nm. The thermal analysis indicated that the P3HT synthesized with the surfactants yielded higher stability and char yields than that of P3HT without. The P3HT_DBSA electrical conductivity was further enhanced by de-doping and doping with HClO_4_. At the 10:1 doping mole ratio, the electrical conductivity of dP3HT_DBSA increased by one order of magnitude relative to P3HT_DBSA prior to the de-doping. The highest electrical conductivity of dP3HT_DBSA obtained was 172 ± 5.21 S cm^−1^ which is the highest value relative to previously reported.

## 1. Introduction

Conductive polymers possess high electrical conductivity through the conjugation system, consisting of the alternate sigma bond and pi bond along the polymer backbone. They have been utilized as sensor materials to detect diverse analytes and biological species [1,2]. Other applications for conductive polymers include as actuators and drug delivery, among many other electronic applications [3].

Conductive polymers can be synthesized by various methods such as chemical, emulsion, electrochemical, photochemical, interfacial, and solid-state polymerizations [4,5,6,7,8,9]. Chemical oxidative polymerization is one of the simplest methods. It is proceeded by the oxidation of monomers with an oxidizing agent, such as ammonium persulfate (APS) or ferric chloride (FeCl_3_), to generate radical cations, and later the coupling between the radicals to generate a conductive polymer. Many conductive polymers have been synthesized by the chemical oxidative polymerization, such as polyacetylene (Pac), poly(p-phenylene) (PPP), polypyrrole (PPy), polythiophene (PTh), and polyaniline (PANI) [10]. The emulsion polymerization is one route into the chemical oxidative polymerization. This synthesis method uses a surfactant to manipulate the particle shape and size and electrical conductivity [5,11]. The polymer particle sizes from the emulsion polymerization have been shown to be in the range of 50–1000 nm [12]. Dodecylbenzenesulfonic acid (DBSA), p-toluenesulfonic acid (PTSA), sodium dodecyl sulfate (SDS), and sodium dioctyl sulfosuccinate (AOT) were the surfactants previously used [13,14,15,16].

Poly(3-hexylthiophene) (P3HT) has been widely studied because of its high charge carrier mobility (>0.1 cm^2^/Vs), ease of synthesis, low cost, good environmental stability [17], and the electrical conductivity values of undoped P3HT were reported to be 2.4 × 10^−6^ S/cm [18], 1.6 × 10^−5^ S/cm [19], 1.22 × 10^−5^ S/cm [20], and 2.6 × 10^−5^ S/cm [21]. The P3HT nanoparticle can be synthesized by emulsion polymerization, in which the polymerization is taking place inside the surfactant micelles [22]. Due to the outstanding properties of P3HT, they have been used in various applications. Khanh et al., 2021, used P3HT as a sensing material to detect ammonia gas in a gas sensor [23]. Tanusorn et al., 2018, used P3HT as a conductive filler to improve the electrical and electromechanical properties of an actuator [24]. Kleinschmidt et al., 2017, used P3HT as a counter electrode in an organic solar cell [25]. Lai et al., 2018, used P3HT as a conductive layer of a positive electrode for lithium-ion batteries [26].

Fukumoto et al., 2013, reported solvent and temperature effects on the regioregularity and polydispersity (PDI) of the P3HT as synthesized by chemical oxidative polymerization. Acetonitrile used as the solvent provided P3HT with a high molecular weight, a large HT content, a narrower PDI and the highest conductivity (3 S cm^−1^) [27]. Duong et al., 2013 investigated efficient p-type doping of P3HT where 7,7,8,8-tetracyano-2,3,5,6-tetrafluoroquinodimethane (F4TCNQ) was used as the dopant. The F4TCNQ dopant increased the P3HT electrical conductivity to 1.82 S cm^−1^ [28].

Presently, there has been no work done on the effects of surfactant type and concentration on the P3HT synthesis to enhance the P3HT electrical conductivity. In the present work, the challenge is to systematically synthesize P3HT by the chemical oxidative emulsion polymerization under various synthesis conditions: monomer; oxidant mole ratios; polymerization times; and surfactant types and concentrations to obtain the highest P3HT conductivity with controllable nanoparticle shape and size. The effect of four anionic surfactants with different structures (AOT, DBSA, SDS, and PTSA) was investigated on the morphology and electrical conductivity of P3HT. It will be shown that the improved P3HT electrical conductivity in this work is mainly derived from the surfactant templates which control the particle shape and size. The synthesized P3HT in the presence of DBSA as the surfactant template is shown here to be potentially used in various electronic applications.

## 2. Materials and Methods

### 2.1. Materials

3-hexathiophene (3HT, 98.0% purity, TCI) was the starting monomer. Ferric chloride (FeCl_3_; 97% purity, Sigma Aldrich, St. Louis, MO, USA) was the oxidant used. Chloroform (CHCl_3_; AR grade, RCI Labscan, Bangkok, Thailand) was the solvent. Dodecylbenzenesulfonic acid (DBSA; 98% purity, Sigma Aldrich), p-toluenesulfonic acid (PTSA; 98.5% purity, Sigma Aldrich), sodium dioctyl sulfosuccinate (AOT; 96% purity, Sigma Aldrich), and sodium dodecyl sulfate (SDS; 98% purity, Sigma Aldrich) were the surfactants. Methanol (99.8%), toluene (99.5%), and hexane (99.8%) were obtained from RCI Labscan. For the de-doping and doping processes, ammonium hydroxide (NH_4_OH; 30% *v*/*v*, Panreac, Barcelona, Spain) was used to de-dope the P3HT_DBSA powder, whereas perchloric acid (HClO_4_; 70% *v*/*v*, Panreac) was the dopant. All reagents were of analytical reagent grades and were used without modification.

### 2.2. Synthesis of Poly(3-hexylthiophene)

The 3HT was the starting monomer, and anhydrous FeCl_3_ and chloroform were the oxidant and solvent, respectively [29]. First, the 3HT monomers were dissolved in 50 mL chloroform. Then, the FeCl_3_ dissolved in 100 mL chloroform was mixed with the monomer solution and the mixture was continuously stirred for 12 h at 40 °C. The oxidizing agent: monomer mole ratios varied from 1:2.5, 1:3, 1:3.5, to 1:4. Finally, the synthesized P3HT was filtered and then rinsed with methanol until the filtrate was colorless, and then it was dried at 80 °C for 24 h. The oxidizing agent to monomer mole ratio of 1:3 was chosen to vary the reaction times to be between 6 h and 24 h.

Surfactants of various types (DBSA, SDS, PTSA, and AOT) were used in the concentrations between 2 CMC to 10 CMC, where CMC is the critical micelle concentration of surfactant. Each surfactant was dissolved in 50 mL of chloroform under continuous stirring at 27 °C for 2 h. The 3HT (0.143 mol, 2.00 g) was added to each surfactant solution. Approximately 5.44 g of FeCl_3_ was dissolved in 150 mL chloroform, where the completely soluble FeCl_3_ in chloroform was observed from the clear solution without precipitation. Then, the FeCl_3_ solution was added to the monomer-surfactant solution and the mixture solution was continuously stirred at 40 °C for 12 h. Next, the obtained polymer was filtrated and subsequently washed several times with methanol until the filtrate was colorless. The filtrate was then dried at 80 °C for 24 h to obtain the P3HT powder. The P3HTs synthesized with the surfactants AOT, DBSA, SDS, and PTSA were coded as P3HT_AOT, P3HT_DBSA, P3HT_SDS, and P3HT_PTSA, respectively.

### 2.3. De-Doping/Doping Process

The P3HT_DBSA at 6 CMC provided the highest electrical conductivity and was further used in the de-doping and the doping processes. The synthesized P3HT_DBSA powder was treated with 2.5 M of NH_4_OH at the $N_{3\mathrm{HT}}$/$N_{{\mathrm{NH}}_{4}\mathrm{OH}}$ mole ratio of 1:10 for 6 h to yield the de-doped P3HT_DBSA (deP3HT_DBSA). The deP3HT_DBSA precipitate was filtered, washed with distilled water, and dried at 80 °C overnight. After that, the deP3HT_DBSA powder was added to a 2.5 M of HClO_4_ solution at the $N_{{\mathrm{HClO}}_{4}}$/$N_{3\mathrm{HT}}$ mole ratios of 1:1, 5:1, 10:1, 25:1, 50:1, and 75:1 for 24 h. The doped P3HT_DBSA (dP3HT_DBSA) was filtered, dried at 80 °C overnight, and kept in a desiccator prior to further use.

### 2.4. Characterizations

The critical micelle concentration (CMC) of each surfactant (DBSA, SDS, PTSA) was determined by a tensiometer (Easydyne K20, Krüss, Hamburg, Germany) at 23 °C. Each surfactant was dissolved in a 100 mL CHCl_3_ solvent. The CMCs of DBSA, SDS, and PTSA were found to be 6.0 × 10^−3^, 5.0 × 10^−3^, and 1.0 × 10^−2^ M, respectively. For AOT, the CMC value used was 6.5 × 10^−3^ as reported by L.D. Giddings and S.V. Olesik, 1994 [30].

The functional groups of P3HT were examined by a Fourier-transform infrared spectrometer (FTIR; Nexus 670, Thermo Scientific, Nicolet iS5, Thermofisher Scientific, Waltham, MA, USA). The P3HT powder was mixed, ground, and pelletized with dried KBr. All P3HT spectra were obtained between 400–4000 cm^−1^, with the 4 cm^−1^ resolution and 64 scans.

The absorption spectra of P3HT were measured by a UV-visible spectrophotometer (UV–Vis; UV-1800, Shimadzu, Tokyo, Japan). 0.0030 g of the P3HTs was dissolved in 10 mL toluene at room temperature. The UV-visible P3HT spectra were recorded between 300–800 nm. The optical band gaps were calculated from the Tauc equation [31].

The crystalline structures of the synthesized P3HT were determined by a wide-angle X-ray diffractometer (XRD, Smartlab, Rigaku, Rigaku Coporation, Tokyo, Japan). The Cu K-alpha radiation source was operated at 40 kv/30 mA, in the 2θ range from 5° to 80°, with a scan speed of 1° min^−1^, and a scan step of 0.02°.

The elemental analysis was carried out by the X-ray photoelectron spectroscopy (XPS; Axis Ultra DLD, Kratos Analytical Ltd., Manchester, UK) using the Al Kα radiation source. The survey scans and high-resolution scans were carried out at the analyzer pass energies of 160 and 40 eV, respectively. A Casa-XPS software (Casa Software Ltd., Las Vegas, NV, USA) was used to analyze the XPS spectra.

The thermal properties of the synthesized P3HT were obtained by a thermal gravimetric analyzer (TGA; TGA 2950, DuPont, Boston, MA, USA). Then, 4–10 mg of each sample was placed in a platinum pan at a heating rate of 10 °C min^−1^ from 30 to 800 °C under a nitrogen atmosphere.

The morphologies, particle shapes and sizes, of the synthesized P3HT were examined by a scanning electron microscope (SEM; S4800, Hitachi, Tokyo, Japan). Each sample was sputtered with a Pt layer of 5 nm before the image measurements. The images were obtained at the magnification of 10k times. The P3HT particle shapes and sizes were analyzed using the SemAfore program (Helsinki, Finland).

The electrical conductivity of synthesized P3HT nanoparticles was measured at ambient temperature by a calibrated two-point probe connected to a power source (Kiethley, 6517A, Portland, OR, USA), as in previous work [16].

## 3. Results and Discussion

### 3.1. Structural Confirmation of the Synthesized P3HT

The P3HT polymerization mechanism is illustrated in Figure S1a (see Supplementary Materials). The 3HT monomers are oxidized to the 3HT radical cations by the oxidant (FeCl_3_). Then, the electrophilic substitution of the radical cations proceeds to obtain the neutral 3HT molecules. In the propagation step, the dimers are coupled and P3HT is generated. The P3HT polymer is then doped by the anion from the dopant as shown in Figure S1b (see Supplementary Materials).

**Figure 1:**
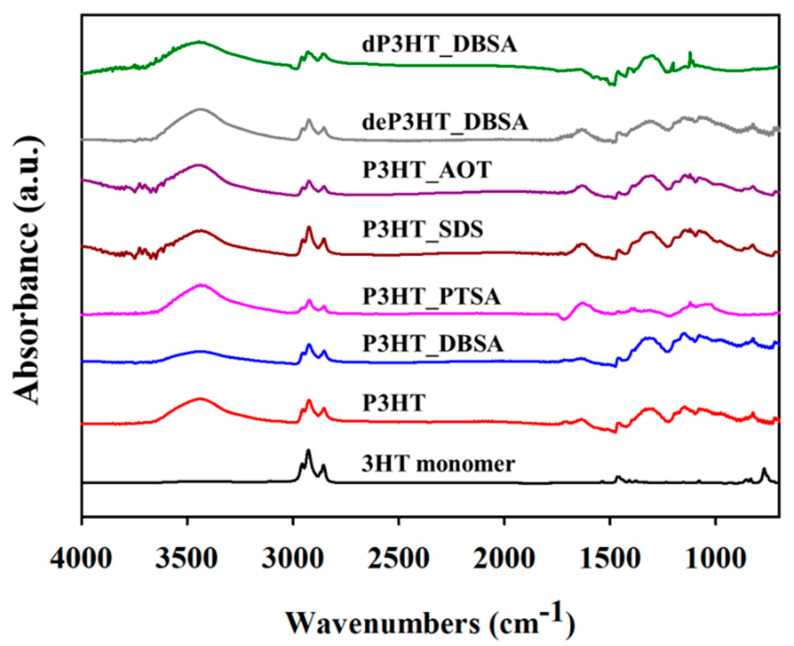
FTIR spectra of P3HT synthesized under different conditions: without surfactant; with various surfactants; before and after the doping process.

The synthesized P3HT structure was verified by FTIR spectra. The FTIR absorption spectra of the P3HT in the presence of DBSA, PTSA, SDS, and AOT at 6 CMC and without a surfactant are shown in Figure 1. The FTIR spectra of all P3HTs display the peaks at 3448 cm^−1^ related to the C–H vibration in the aliphatic chain of hexyl groups [32], 1581–1444 cm^−1^ and 822 cm^−1^ corresponding to the symmetric and asymmetric C=C ring stretching vibration of the thiophene ring, and the C–H out-of-plane vibration of a 2,3,5-trisubstituted ring, respectively [33]. The hexyl side chains of the P3HT are confirmed from the band between 2924–2853 cm^−1^. The peaks at 1392 cm^−1^ and 718 cm^−1^ are due to the -CH_2_ asymmetric and symmetric stretching of the aliphatic side chain and the –CH_3_ symmetric bending from the terminal methyl groups –CH_3_ and the hexyl substituent methylene group –(CH_2_)_5_– in phase rocking, respectively [33,34]. The observed FTIR spectra confirm the successful P3HTs synthesis. For the P3HT synthesized with the surfactants, the peak at 1045 cm^−1^ belongs to the sulfur atom of the S=O vibrations of DBSA and SDS [35,36]. The peak at 1734 cm^−1^ indicates the C=O vibration of AOT [37]. The peak at 1435 cm^−1^ represents the methyl C–H asymmetric bending of PTSA [38]. The FTIR spectra of the different surfactants confirm that the surfactants were present in the P3HT structure.

FTIR results confirm that the surfactants can be observed in the polymer backbone, indicating that the removal of surfactants was not complete as they tend to permanently accumulate on the surface of the nanostructures, resulting ultimately in hindering or blocking ion exchange and impairing electroactivity. Therefore, the synthesized polymer may not be promising to use in electrochemical applications. The synthesized polymer is, however, better used, in a powder form, in certain applications such as drug delivery and as an actuator [2,3].

### 3.2. UV-Vis Spectroscopy

**Figure 2:**
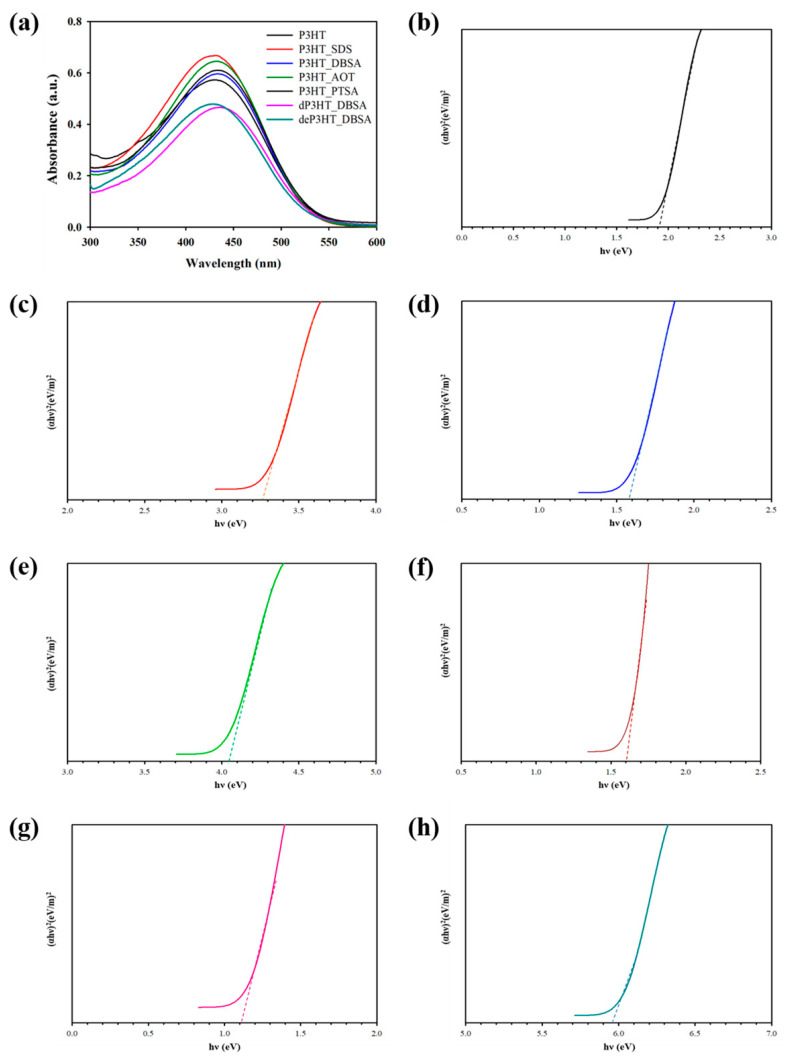
UV-vis absorption spectra and Tauc plots of P3HT: (**a**) UV–vis absorption spectra of P3HT synthesized under different conditions; (**b**) Tauc plot of P3HT; (**c**) Tauc plot of P3HT_SDS; (**d**) Tauc plot of P3HT_DBSA; (**e**) Tauc plot of P3HT_AOT; (**f**) Tauc plot of P3HT_PTSA; (**g**) Tauc plot of dP3HT_DBSA; and (**h**) Tauc plot of deP3HT_DBSA.

Figure 2 illustrates the UV-vis spectra of the synthesized P3HT without a surfactant and with different surfactants all at 6 CMC. The synthesized P3HT spectra display the apparent UV-vis peaks between 434–437 nm. For the P3HT synthesized without a surfactant, the UV-vis peak at the wavelength (λ_max_) at around 434 nm indicates the π–π* transition of the thiophene rings [39]. For the P3HT with the surfactants, the λ_max_ values of the P3HT_DBSA, P3HT_AOT, and P3HT_PTSA are at 437 nm, 435 nm, and 437 nm, respectively. The UV–vis peaks shift to longer wavelengths (red shift) due to electron delocalization, reflecting the molecular interactions between the polymer and the surfactants [40]. Moreover, the peak shifts to higher wavelengths can be referred to as the smaller band gaps in accordance with the Tauc relation [41]. However, the UV–vis peak shift of P3HT_SDS was not found (434 nm).

The optical band gap value of P3HT synthesized without a surfactant is 1.90 eV, consistent with the previous result [42]. At 6 CMC, the P3HT_DBSA provides the narrowest optical band gap value (1.56 eV), followed by P3HT_PTSA (1.60 eV), P3HT_SDS (3.25 eV), and P3HT_AOT (4.05 eV), respectively. The decrease in the optical band gaps might occur from the surfactants acting as the dopant molecules, leading to the electrons in HOMO being easily promoted to LUMO [43]. Therefore, the shift of the UV-Vis peak and the narrowest optical band gap energy of the P3HT backbone occurs from the interaction between the P3HT and surfactants [40].

The UV–vis absorption, originating from the π–π* transition in the presumably neutral state, is observed even though the doping levels of these polymers are 7–26% from the XPS. This is because P3HT was slightly doped from Cl^−^ from the FeCl_3_. Herein, the polaron and bipolaron peaks cannot be measured by UV–vis spectroscopy because the UV spectrometer (Shimadzu, UV-1800) can only be measured in wavelengths between 300–800 nm. However, the polaron and bi-polaron states are located at about 1600 nm and 920 nm, respectively [44].

### 3.3. X-ray Diffraction

**Figure 3:**
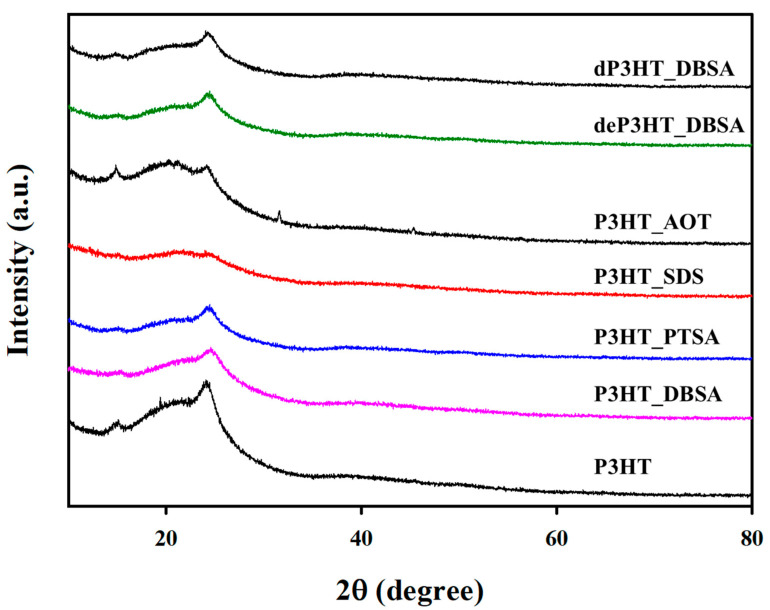
XRD patterns of P3HT synthesized under different conditions: without surfactants; with various surfactants; and before and after the doping process.

Figure 3 shows the XRD patterns of the P3HT synthesized without and with the surfactants. The P3HT synthesized without a surfactant exhibits a strong peak at 2θ = 23.8° and a weak peak at 2θ = 10.7°, suggesting the orthorhombic structures as represented by the (010) and (200) planes with the plane spacings: d(010) = 3.8 Ǻ, and d(200) = 8.25 Ǻ, respectively [45,46]. The peak intensities at 10.7°, and 23.8° decrease under the presence of the surfactants, indicating the loss of crystallinity with the surfactants, consistent with previous work [46]. However, the diffraction peak at 2θ ~ 10.7° of AOT is clearly observed, whereas it is weak for other surfactants. This might have resulted from the steric hindrance from the two tails of the AOT molecules, preventing the incorporation of AOT molecules between P3HT chains.

### 3.4. X-ray Photoelectron Spectrometer

XPS analysis was utilized here to determine individual elements and the degree of doping on the sample surface [16,47,48,49]. The wide scan XPS spectra of the synthesized P3HTs of various surfactants are shown in Table S1 (see Supplementary Materials); they do not show a peak of Fe at 720 eV. Therefore, Fe was not incorporated into the polymer backbone. This confirms that Cl^−^ is truly the dopant, without a trace of FeCl_3_. The doping level can be determined by the contents of chloride ions (Cl^−^) and sulfur cations (–S^+^=); the doping levels were calculated by the ratios of the chloride ions to sulfur content (Cl/S) [37,38,39,40], and the data are tabulated in Table S1 (see Supplementary Materials).

For the synthesized P3HTs without a surfactant, as shown in Table S1 (see Supplementary Materials), the doping levels of P3HT_1:2.5, P3HT_1:3, P3HT_1:3.5, and P3HT_1:4 at the polymerization time of 12 h are 7.8%, 26.0%, 8.9%, and 7.3%, respectively. Therefore, P3HT_1:3 possesses the highest doping level. Therefore, P3HT_1:3 at the polymerization time of 12 h was used in further studies.

For the synthesized P3HTs with the four surfactants at 6 CMC, at the 3HT: FeCl_3_ mole ratio of 1:3 and at the polymerization time 12 hr, the doping levels are shown in Table S1 (see Supplementary Materials). The P3HT_DBSA has the highest doping level of 21.6%, higher than those of PTSA (19.0%), SDS (14.2%), and AOT (12.5%). The highest doping level of P3HT_DBSA suggests that DBSA has the highest doping efficiency. This may result from the π−π interaction between the DBSA benzene rings screening the electrostatic repulsion between the DBSA head groups, resulting in the higher probability of Cl^−^ getting into the micelle to incorporate within the P3HT chains.

A comparison of the doping levels of the P3HTs synthesized with a DBSA at various CMCs is shown in Table S1 (see Supplementary Materials). P3HT_DBSA at 6 CMC exhibits the highest doping level (21.55%). Therefore, P3HT_DBSA (6 CMC) was investigated further.

### 3.5. Thermal Gravimetric Analysis

**Figure 4:**
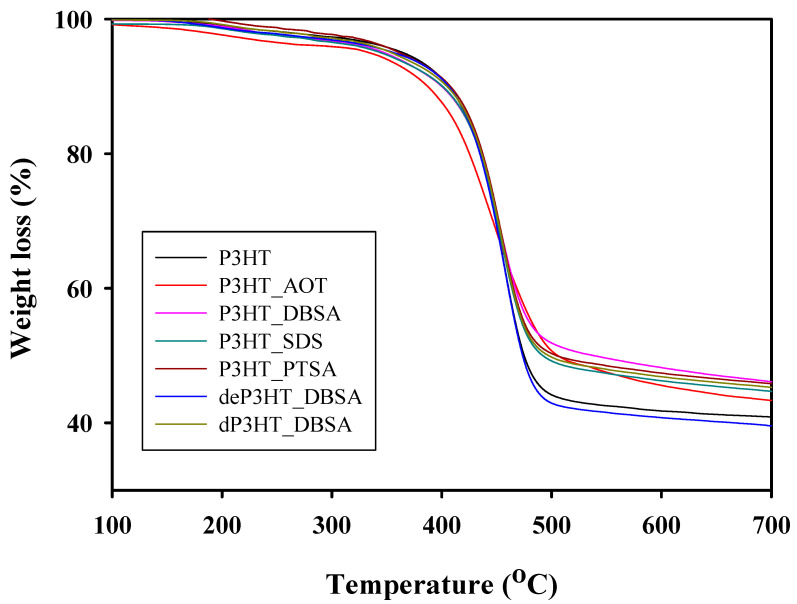
TGA thermograms of P3HT synthesized by chemical oxidative polymerization without and with various surfactants.

The thermal properties of the synthesized P3HTs without a surfactant and with various surfactants (AOT, DBSA, SDS, PTSA) at 6 CMC were investigated by TGA as shown in Figure 4. All five samples show one-step decomposition temperatures. The onset decomposition temperatures (T_d, onset_) of P3HT, P3HT_AOT, P3HT_DBSA, P3HT_SDS and P3HT_PTSA are 413 °C, 417 °C, 419 °C, 417 °C, and 418 °C, respectively, consistent with the previous work [34]. The P3HT with the surfactants show higher T_d_, _onset_ than the P3HT without a surfactant. The results suggest that the T_d, onset_ increases from the presence of the surfactants, due to the differences in particle shapes and surface areas for the thermal transport. T_d, onset_ of P3HT_DBSA (6 CMC) is the highest because its shape is the sphere cluster (grape shape) with the highest packing density than the irregular shape [13]. Therefore, the P3HT_DBSA (6 CMC) has better thermal stability than other synthesis conditions. Moreover, the residue increases with increasing T_d, onset_ as a higher T_d, onset_ corresponds to a shorter decomposition time. The residues of the polymeric chains are 39.38%, 42.07%, 43.62%, 43.45%, and 43.55%, respectively.

### 3.6. Morphology

**Figure 5:**
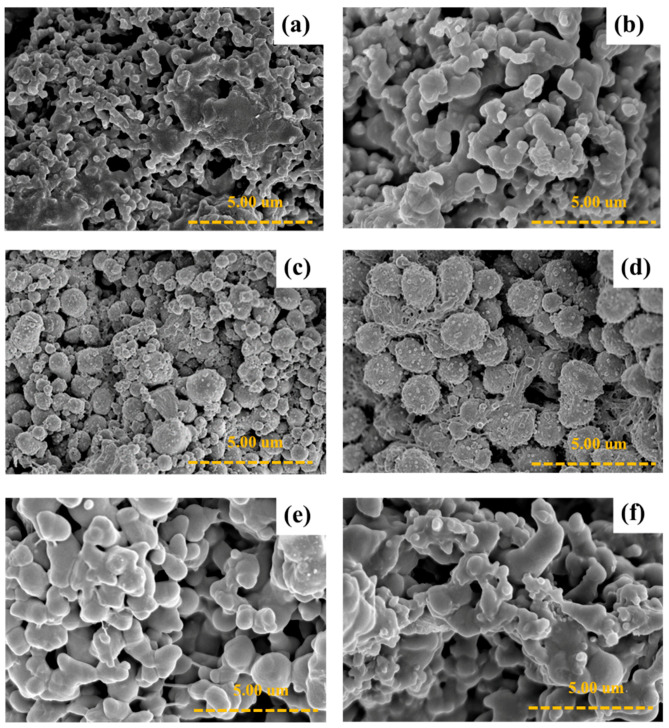
SEM images of P3HT synthesized by chemical oxidative polymerization: (**a**) without DBSA; with various DBSA concentrations (**b**) P3HT_DBSA at 2 CMC; (**c**) P3HT_DBSA at 4 CMC; (**d**) P3HT_DBSA at 6 CMC; (**e**) P3HT_DBSA at 8 CMC; and (**f**) P3HT_DBSA at 10 CMC.

Figure 5 shows the morphologies of the P3HT without a surfactant and P3HTs of various DBSA concentrations. For the P3HT synthesized without a surfactant (Figure 5a), the formation of the P3HT displays an irregular shape due to having no micelles to stabilize the particles [5]. For the morphologies of the synthesized P3HTs of various DBSA concentrations (Figure 5b–f), the particle shape changes from an irregular shape (without a surfactant) to a sphere cluster (grape shape). The particle shape tends to become more spherical with an increasing DBSA concentration. At 6 CMC, the particle shape is spherical, connected with some fibers like a grape shape, similar to previous work [50]. Upon increasing the auto-dopant level, the morphology of the P3HT changed from a granular structure to a fibrillar structure. As more polarons and bipolarons were generated, they induced a granule-to-nanowire transition [50]. Beyond 6 CMC, the particle shape is a sphere cluster (a fusion of spheres) with smaller spherical particle sizes. The reduction in the spherical particle sizes is due to the dense micelle formation from the micelles at high concentrations [50,51]. The particle sizes and shapes of the P3HT synthesized with various DBSA concentrations are tabulated in Table 1. The morphologies of P3HT under other surfactant types namely PTSA, SDS, and AOT are reported in Figures S2–S4 (see Supplementary Materials).

### 3.7. Electrical Conductivity

**Figure 6:**
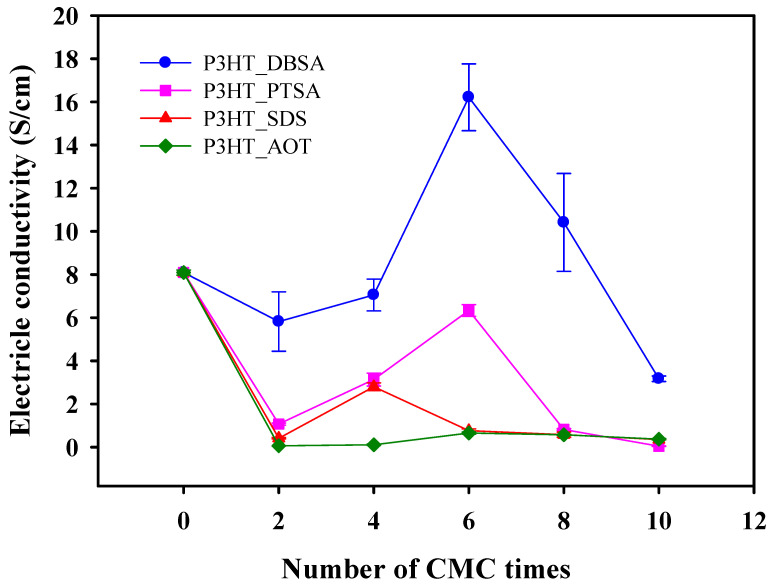
Electrical conductivity of P3HT synthesized by chemical oxidative polymerization with various surfactants at the monomer to oxidant ratio of 1:3 and at the polymerization time of 12 h.

The electrical conductivity values vs. surfactant concentration are shown in Figure 6. The particle sizes and shapes and electrical conductivity values of all P3HTs synthesized here are tabulated in Table 1. The P3HT without a surfactant possesses the electrical conductivity of 8.09 ± 0.12 S cm^−1^ with a particle size of 141 ± 21 nm. In the comparison between the P3HT synthesized without a surfactant and with different surfactants at 6 CMC, the P3HT_DBSA possesses a higher electrical conductivity value (16.21 ± 1.55 S cm^−1^) than those of P3HT, P3HT_PTSA, P3HT_SDS, and P3HT_AOT. Even though the chemical structures of DBSA and PTSA are similar to the sulfonate groups (SO_3_^2−^) and the benzene rings in their head groups, the electrical conductivity value with the largest particle size of P3HT_DBSA is higher. Generally, the electrical conductivity increases with decreasing particle size due to the quantum size effect [52]. The explanation for the higher P3HT_DBSA electrical conductivity with the largest particle size may be related to the fact that the DBSA molecule has a longer tail than PTSA. Therefore, DBSA interacts with a lesser degree with P3HT than PTSA, thus promoting the higher dopant Cl^−^ interaction with P3HT, as shown by the highest doping level of P3HT_DBSA from XPS.

Therefore, the electrical conductivity values of P3HT, as supported by the PTSA, SDS, and AOT, are lower than the pure P3HT because these surfactants hinder the interaction between the chloride ion (Cl^−^) of the oxidant, acting as one of the dopants, and the P3HT backbone. This explanation is confirmed by the decrease in Cl *2p* (% At) in Table S2 (see Supplementary Materials). Therefore, PTSA, SDS, and AOT produce the lowest numbers of charge carriers (Cl^−^ ions) in the P3HT backbone.

Under the effect of DBSA concentrations, the electrical conductivity values of P3HT_DBSA for the DBSA concentrations of 2 CMC, 4 CMC, 6 CMC, 8 CMC, and 10 CMC are 5.82 ± 1.38 S cm^−1^, 7.05 ± 0.74 S cm^−1^, 16.21 ± 1.55 S cm^−1^, 10.41 ± 2.27 S cm^−1^, and 3.17 ± 0.13 S cm^−1^, respectively. The highest electrical conductivity of P3HT_DBSA is 16.21 ± 1.55 S cm^−1^ at the DBSA concentration of 6 CMC. The possible reasons for the high P3HT_DBSA electrical conductivity are: DBSA acts as a dopant [53] the π–π interaction between the DBSA benzene rings and the P3HT chain; or the remaining DBSA molecules attached to the P3HT backbone can also act as a dopant, as shown by the present XPS and FTIR results [13]. At higher surfactant concentrations above 6 CMC, a decrease in the electrical conductivity is due to the self-repulsion of surfactant head-group ions during the polymerizing and doping processes and, thus, the denser micelle formation, leading to the lesser penetration of Cl^−^ into micelles to interact with P3HT chains. This finding, therefore, is consistent with the reduction in the doping level at high DBSA concentrations as shown in Table S1 (see Supplementary Materials).

### 3.8. De-Doping/Doping Process

The P3HT_DBSA (6 CMC) powder was dispersed into the strong base (NH_4_OH) at room temperature to yield the de-doped P3HT and deP3HT_DBSA. The de-doping process was verified by UV–vis spectroscopy and XPS. The optical band gap value of P3HT_DBSA before and after the de-doping process increased from 1.56 eV to 5.95 eV, respectively. This occurred from the removal of the dopant (Cl^−^ ions) from P3HT_DBSA. The survey scan XPS spectrum of deP3HT_DBSA indicated a disappearance of Cl 2p, consistent with the optical band gap value of deP3HT_DBSA. This result suggests that P3HT_DBSA was de-doped successfully. The electrical conductivity of P3HT_DBSA decreased from 16.21 ± 1.55 S cm^−1^ to 2.01 × 10^−5^ S cm^−1^, a decrease of six orders of magnitude.

For the re-doping process with HClO_4_, the FTIR spectra of the dP3HT_DBSA consisted of several peaks of the ClO_4_ vibration at 1061–1089 cm^−1^, 1104–1108 cm^−1^, and 1121–1145 cm^−1^, suggesting that the doping of P3HT_DBSA was successful [36].

**Figure 7:**
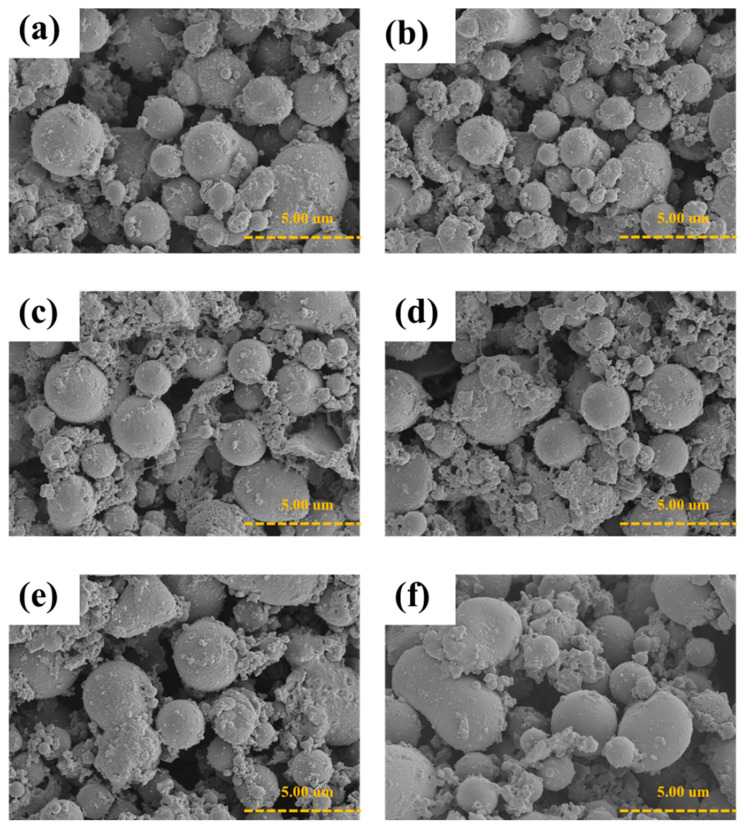
SEM images of HClO_4_ doped P3HT_DBSA synthesized at various doping levels: (**a**) dP3HT_DBSA (1:1); (**b**) dP3HT_DBSA (5:1); (**c**) dP3HT_DBSA (10:1); (**d**) dP3HT_DBSA (25:1); (**e**) dP3HT_DBSA (50:1); and (**f**) dP3HT_DBSA (75:1).

The SEM images of the P3HT_DBSA of various doping mole ratios are shown in Figure 7; the particle shape remains spherical. No difference in the P3HT_DBSA particle shape is noticeable for the P3HT_DBSA of different HClO_4_ mole ratios. The particle shapes were similar before and after doping.

**Figure 8:**
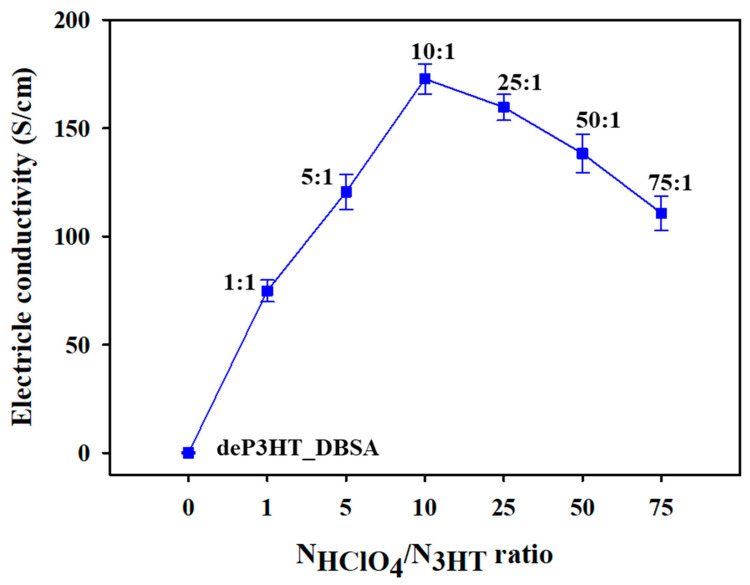
Electrical conductivity of the de-doped P3HT_DBSA and HClO_4_ doped P3HT_DBSA at different $N_{{\mathrm{HClO}}_{4}}$/$N_{3\mathrm{HT}}$ doping mole ratios.

Figure 8 shows the electrical conductivity of the doped P3HT_DBSA in terms of the $N_{{\mathrm{HClO}}_{4}}$/$N_{3\mathrm{HT}}$ mole ratio. The highest dP3HT_DBSA electrical conductivity is obtained from the doping mole ratio of 10:1 (172.74 ± 5.21 S cm^−1^); it is higher by 1 order of magnitude relative to the P3HT_DBSA before the de-doping (16.21 ± 1.55 S cm^−1^). The increase stems mainly from the increase in the number of charge carriers, namely the polaron and bipolaron species [16]. However, the electrical conductivity values at the doping mole ratios higher than 10:1 become smaller as shown in Figure 8. This is simply due to the excessive dopant amounts generating the electrostatic repulsion resulting in the incomplete doping process, as confirmed by the reduction in doping level from XPS (Table S2, see Supplementary Materials) and the increase in the optical band gap energy from UV–vis spectroscopy (Table S3, see Supplementary Materials). The XRD patterns of deP3HT_DBSA and dP3HT_DBSA are nearly the same patterns as the P3HT_DBSA pattern as shown in Figure 3.

The P3HT shape, particle size, and electrical conductivity values at different synthesis conditions of the present work and of previous reports are summarized in Table 1. Karim, (2012), synthesized a soluble P3HT/silanized MWNT composite by in situ chemical oxidative polymerization [54]. The SEM image of P3HT/MWNT showed the well-dispersed MWNT in the polymer matrix in tubular structures, and the conductivity values of the P3HT homopolymer and P3HT/MWNT composite were 2.3 × 10^−5^ S cm^−1^ and 0.71 S cm^−1^, respectively [54]. Du et al., (2012), prepared P3HT/GNs composites by a chemical oxidative polymerization method using chloroform as a solvent and FeCl_3_ as an oxidant. The highest electrical conductivity of P3HT/GNs composites was 1.2 S cm^−1^ at 30 wt% of GNs [40]. Fukumoto et al., (2013), reported solvent and temperature effects on regioregularity and polydispersity (PDI) of the P3HT synthesized by chemical oxidative polymerization (COP). The CH_3_CN provided P3HT with a high molecular weight, a large HT content, a narrower PDI, and the highest conductivity (3 S cm^−1^) [27]. Duong et al., (2013), investigated the efficient p-type doping on P3HT where 7,7,8,8-tetracyano-2,3,5,6-tetrafluoroquinodimethane (F4TCNQ) was used as a dopant. The added F4TCQN increased the in-plane conductivity of P3HT films to 1.82 S cm^−1^ [28].

In this work, P3HT synthesized by chemical oxidative polymerization at the FeCl_3_ concentration of 2.37 mol L^−1^ and the 3HT: FeCl_3_ mole ratio of 1:3 provided a high electrical conductivity of 8.09 ± 0.12 S cm^−1^ with an irregular shape and with the particle size of 141 ± 21 nm. The high electrical conductivity of P3HT obtained in the presence of DBSA was 16.21 ± 1.55 S cm^−1^, where the P3HT particle shapes were of a spherical grape shape with an average size of 1526 ± 227 nm. At the re-doping mole ratio of 10:1, the electrical conductivity of dP3HT_DBSA increased by 1 order of magnitude relative to the P3HT_DBSA before de-doping. In this work, the highest electrical conductivity of dP3HT_DBSA obtained is as high as 172.74 ± 5.21 S cm^−1^.

### 3.9. The Correlation of Electrical Conductivity and Doping Level

**Figure 9:**
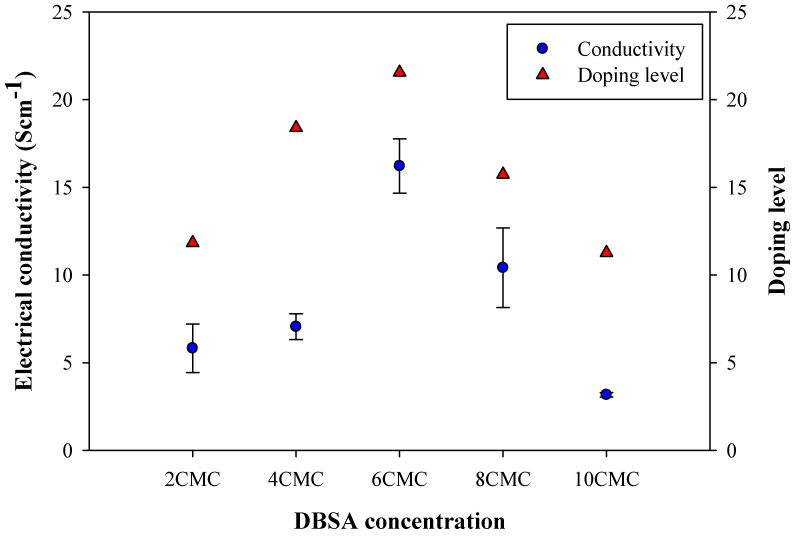
The relationship between the electrical conductivity and the doping level of P3HT at various surfactant concentrations.

The electrical conductivity and the doping level of P3HT at various surfactant concentrations are shown in Figure 9. The electrical conductivity of P3HT (at 0 CMC) without a surfactant is 8.09 ± 0.12 S cm^−1^. The electrical conductivity (the % doping level) of P3HT_DBSA at 2 CMC, 4 CMC, 6 CMC, 8 CMC, and 10 CMC are 5.82 ± 1.38 (11.8%), 7.05 ± 0.74 (18.4%), 16.21 ± 1.55 (21.6%), 10.41 ± 2.27 (15.7%), and 3.17 ± 0.13 (11.3%) S cm^−1^, respectively. The electrical conductivity can be seen to be well correlated with the doping level, as shown in Figure 9. The electrical conductivity and doping level of P3HT tend to increase with the increasing DBSA concentration from 2 CMC to 6 CMC, then they decrease above 6 CMC DBSA.

DBSA acts as the dopant due to the π–π interaction between the DBSA benzene rings and the P3HT chain [13]. At a higher DBSA concentration between 2 CMC and 6 CMC, the electrical conductivity and the doping level increase due to the π–π interaction between the DBSA benzene rings and the P3HT chain, and the CI^−^ from the oxidant was incorporated to the P3HT within the micelle [16]. However, above the DBSA concentration of 6 CMC, the electrical conductivity and the doping level decrease because of the self-repulsion of DBSA groups leading to the lesser interaction between Cl^−^ and the P3HT within the micelle [16]. The incorporation of CI^−^ is confirmed by XPS in Table S1 (see Supplementary Materials, effect of critical micelle concentrations at the 3HT: FeCl_3_ mole ratio of 1:3 in DBSA).

**Figure 10:**
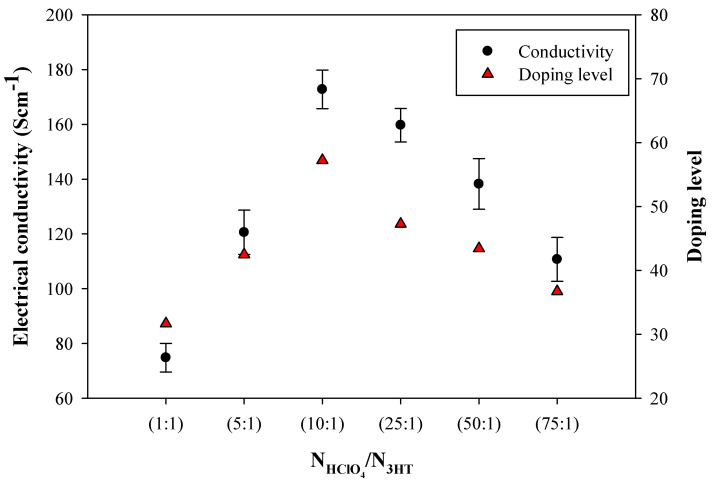
The relationship between the electrical conductivity and the doping level of P3HT (At the DBSA concentration of 6 CMC) at various $N_{{\mathrm{HClO}}_{4}}$/$N_{3\mathrm{HT}}$ mole ratios.

The electrical conductivity and the doping level of P3HT (at the DBSA concentration of 6 CMC) at various $N_{{\mathrm{HClO}}_{4}}$/$N_{3\mathrm{HT}}$ mole ratios are shown in Figure 10. The electrical conductivity of deP3HT_DBSA is 2.01 × 10^−5^ S cm^−1^. The electrical conductivity (the % doping level = Cl/S) of the doped P3HT_DBSA at the 1:1, 5:1, 10:1, 25:1, 50:1, and 75:1 $N_{{\mathrm{HClO}}_{4}}$/$N_{3\mathrm{HT}}$ mole ratios are 74.81 ± 5.22 (31.7%), 120.56 ± 8.10 (42.5%), 172.74 ± 7.02 (57.2%), 159.68 ± 6.11 (47.3%), 138.24 ± 9.25 (43.4%), and 110.70 ± 8.01 (36.7%) S cm^−1^, respectively. At a higher $N_{{\mathrm{HClO}}_{4}}$/$N_{3\mathrm{HT}}$ mole ratio (1:1 to 10:1), the electrical conductivity and the doping level tend to increase because a higher amount of Cl^−^ ion interacted with the P3HT chain, leading to an increase in the number of charge carriers [16]. Above the $N_{{\mathrm{HClO}}_{4}}$/$N_{3\mathrm{HT}}$ 10:1 mole ratio, the electrical conductivity and the doping level decrease because the excessive dopant amounts generated the electrostatic repulsion resulting in the incomplete doping process [36].

## 4. Conclusions

Poly(3-hexylthiophene) (P3HT) was successfully prepared by chemical oxidative polymerization of 3HT with ferric chloride (FeCl_3_) as the oxidizing agent, with and without surfactants. After that, dodecylbenzenesulfonic acid (DBSA), p-toluenesulfonic acid (PTSA), sodium dodecyl sulfate (SDS), and sodium dioctyl sulfosuccinate (AOT) were utilized as the surfactant templates to investigate the influences of surfactant types and concentrations in the P3HT synthesis. The structures of P3HT with and without surfactants were identified by the FTIR and XRD analysis. For the effects of surfactant type and concentration, the P3HT prepared with DBSA at 6 CMC provided higher electrical conductivity than those in PTSA, SDS, and AOT. The highest electrical conductivity of P3HT obtained in the presence of DBSA was 16.21 ± 1.55 S cm^−1^ with the lowest energy gap of 1.56 eV where the P3HT particle shapes were spherical (grape shape), with the average size of 1526 ± 227 nm. The thermal stability of the P3HT investigated showed that the P3HTs synthesized with the surfactants were of higher thermal stability than the P3HT without a surfactant. The highest electrical conductivity of P3HT_DBSA was improved by de-doping and doping with HClO_4_ at the doping mole ratio of 10:1, and the electrical conductivity of dP3HT_DBSA was improved by 1 order of magnitude relative to the P3HT_DBSA before de-doping. The highest electrical conductivity of dP3HT_DBSA obtained was 172.74 ± 5.21 S cm^−1^. Therefore, the synthesized P3HT with DBSA as the surfactant template has the potential to be developed and applied further in various electronic applications, such as sensors and actuators.

## Figures and Tables

**Table 1:** Comparison of particle size, particle shape, and electrical conductivity of P3HT with previous works.

Synthesis Method	Surfactant Type	Particle Size (nm)	Particle Shape	Electrical Conductivity (S cm^−1^)	Sample Code	Ref.
Chemical oxidative polymerization	-	157 ± 33	Irregular	3.12 ± 0.44	P3HT at 6 h	-
	-	141 ± 21	Irregular	8.09 ± 0.12	P3HT at 12 h	-
	-	280 ± 62	Agglomerated	3.04 ± 0.43	P3HT at 18 h	-
	-	311 ± 69	Agglomerated	2.33 ± 0.20	P3HT at 24 h	-
	-	650 ± 197	Irregular	0.24 ± 0.05	3HT:FeCl_3_ = 1:2.5	-
	-	141 ± 21	Irregular	8.09 ± 0.12	3HT:FeCl_3_ = 1:3	-
	-	191 ± 48	Agglomerated	1.23 ± 0.36	3HT:FeCl_3_ = 1:3.5	-
	-	347 ± 139	Agglomerated	0.49 ± 0.12	3HT:FeCl_3_ = 1:4	-
Emulsion polymerization	DBSA	244 ± 58	Sphere cluster and fiber	5.82 ± 1.38	P3HT_DBSA 2 CMC	-
		542 ± 139	Spherical	7.05 ± 0.74	P3HT_DBSA 4 CMC	-
		1526 ± 227	Grape shape	16.21 ± 1.55	P3HT_DBSA 6 CMC	-
		672 ± 192	Sphere cluster	10.41 ± 2.27	P3HT_DBSA 8 CMC	-
		516 ± 131	Sphere cluster and fiber	3.17 ± 0.13	P3HT_DBSA 10 CMC	-
	PTSA	356 ± 65	Irregular	1.07 ± 0.05	P3HT_PTSA 2 CMC	-
		316 ±82	Irregular	3.13 ± 0.30	P3HT_PTSA 4 CMC	-
		254 ± 58	Coral reef shape	6.33 ± 0.27	P3HT_PTSA 6 CMC	-
		364 ± 118	Fused spheres and fiber	0.82 ± 0.04	P3HT_PTSA 8 CMC	-
		414 ± 70	Fused spheres and fiber	0.04 ± 0.01	P3HT_PTSA 10 CMC	-
	SDS	247 ± 38	Spherical	0.41 ± 0.03	P3HT_SDS 2 CMC	-
		234 ± 48	Spherical	2.80 ± 0.18	P3HT_SDS 4 CMC	-
		300 ± 49	Spherical	0.76 ± 0.08	P3HT_SDS 6 CMC	-
		498 ± 98	Sphere cluster	0.58 ± 0.12	P3HT_SDS 8 CMC	-
		817 ± 54	Sphere cluster	0.35 ± 0.02	P3HT_SDS 10 CMC	-
	AOT	348 ± 92	Spherical	0.06 ± 0.01	P3HT_AOT 2 CMC	-
		335 ± 97	Spherical and fiber	0.11 ± 0.03	P3HT_AOT 4 CMC	-
		269 ± 98	Spherical and fiber	0.65 ± 0.05	P3HT_AOT 6 CMC	-
		278 ± 73	Spherical and fiber	0.57 ± 0.02	P3HT_AOT 8 CMC	-
		291 ± 53	Spherical and fiber	0.37 ± 0.03	P3HT_AOT 10 CMC	-
De-doping with NH_4_OH	-	1545 ± 861	Spherical	2.01 × 10^−5^	deP3HT_DBSA	-
Doped with HClO_4_	-	1223 ± 718	Spherical	74.81 ± 5.22	dP3HT_DBSA (1:1)	-
	-	1232 ± 463	Spherical	120.56 ± 8.10	dP3HT_DBSA (5:1)	-
	-	1110 ± 494	Spherical	172.74 ± 7.02	dP3HT_DBSA (10:1)	-
	-	1389 ± 873	Spherical	159.68 ± 6.11	dP3HT_DBSA (25:1)	-
	-	1170 ± 654	Spherical	138.24 ± 9.25	dP3HT_DBSA (50:1)	-
	-	1530 ± 827	Spherical	110.70 ± 8.01	dP3HT_DBSA (75:1)	-
In situ polymerization	-	-	Agglomerated	2.30 × 10^−5^	P3HT	[48]
	-	-	Tubular	0.71	P3HT_MWNT	[48]
Chemical oxidative polymerization	-	-	Agglomerated	1.20	P3HT_GNs	[34]
	-	-	-	3.00	P3HT	[21]
	-	-	-	1.82 ± 0.22	P3HT doped with F4TCNQ	[22]

## Data Availability

The data presented in this study are available on request from the corresponding author. The data are not publicly available due to author’s readiness to provide it on request.

## Supplementary Materials

The following supporting information can be downloaded at: https://www.mdpi.com/article/10.3390/polym14183860/s1, Figure S1: Proposed polymerization mechanism of P3HT: (a) polymerization process; (b) doping process; Figure S2: SEM images of P3HT synthesized by chemical oxidative polymerization with various PTSA concentrations: (a) P3HT_PTSA at 2 CMC; (b) P3HT_PTSA at 4 CMC; (c) P3HT_PTSA at 6 CMC; (d) P3HT_PTSA at 8 CMC; and (e) P3HT_PTSA at 10 CMC; Figure S3: SEM images of P3HT synthesized by chemical oxidative polymerization with various SDS concentrations: (a) P3HT_SDS at 2 CMC; (b) P3HT_SDS at 4 CMC; (c) P3HT_SDS at 6 CMC; (d) P3HT_SDS at 8 CMC; and (e) P3HT_SDS at 10 CMC; Figure S4: SEM images of P3HT synthesized by chemical oxidative polymerization with various AOT concentrations: (a) P3HT_AOT at 2 CMC; (b) P3HT_AOT at 4 CMC; (c) P3HT_AOT at 6 CMC; (d) P3HT_AOT at 8 CMC; and (e) P3HT_AOT at 10 CMC; Table S1: XPS data of P3HT synthesized with and without surfactant at various oxidant: mole ratios at the polymerization time of 12 h; Table S2: XPS data of P3HT synthesized at various doping levels; Table S3: Optical band gaps of the deP3HT, dP3HT_DBSA (1:1), dP3HT_DBSA (5:1), dP3HT_DBSA (10:1), dP3HT_DBSA (25:1), dP3HT_DBSA (50:1), and dP3HT_DBSA (75:1); The calculation of optical band gap energy. Reference [55] are cited in the supplementary materials.
