# Supplementary material for: Synthesis of Highly Conductive Poly(3-hexylthiophene) by Chemical Oxidative Polymerization Using Surfactant Templates
Source: Polymers (Basel). 2022 Sep 15;14(18):3860. doi: 10.3390/polym14183860 (PMC9503232; doi:10.3390/polym14183860)
Supplement: Supplementary file 1 [file polymers-14-03860-s001.zip › polymers-1874026-supplementary.pdf]

Synthesis of Highly Conductive Poly(3-hexylthiophene) by Chemical Oxidative Polymerization Using Surfactant Templates

Sanhanut Kesornsit [1], Chatrawee Direksilp [1,2], Katesara Phasuksom [1,2], Natlita Thummarungsan [1,2], Phimchanok Sakunpongpitiporn [1], Kornkanok Rotjanasuworapong [1], Anuvat Sirivat [1,2,*] and Sumonman Niamlang [3,*]

1 Conductive and Electroactive Polymers Research Unit, The Petroleum and Petrochemical College, Chulalongkorn University, Bangkok 10330, Thailand;

2 Center of Excellence on Petrochemical and Materials Technology (PETROMAT), Chulalongkorn University Research Building, Soi Chula 12, Phayathai Road, Bangkok 10330, Thailand

3 Department of Materials and Metallurgical Engineering, Faculty of Engineering, Rajamangala University of Technology Thanyaburi, Pathumthani 12110, Thailand

* Correspondence: anuvat.s@chula.ac.th (A.S.); sumonman.n@en.rmutt.ac.th (S.N.); Tel.: +662-218-4131 (A.S.); Fax: +662-611-7221 (A.S.)

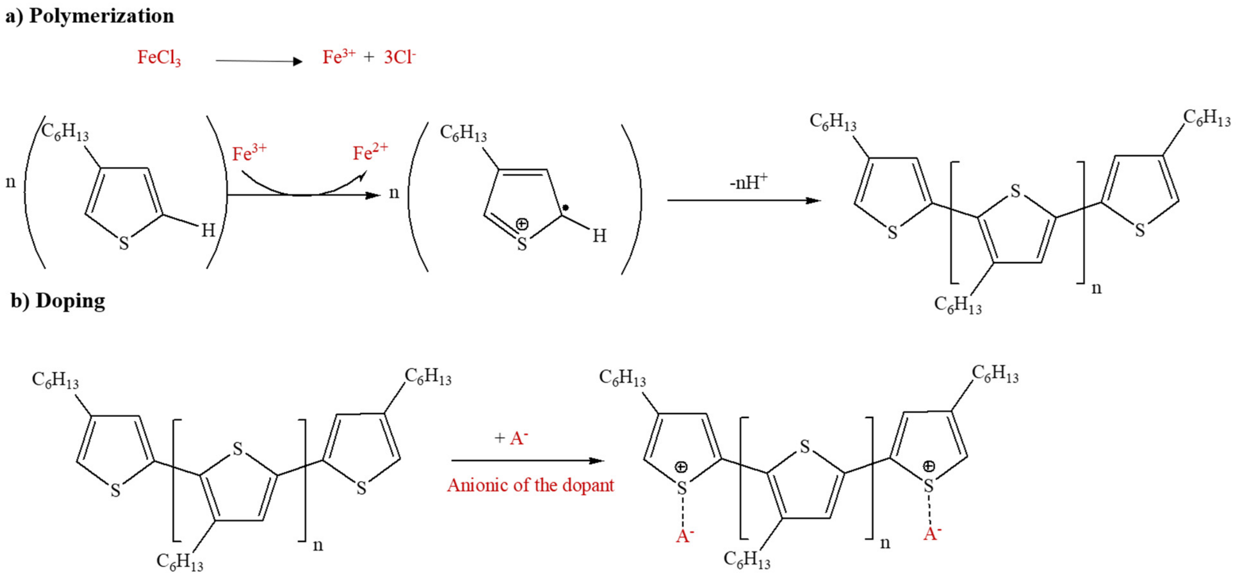

Figure S1. Proposed polymerization mechanism of P3HT: (a) polymerization process; (b) doping process.

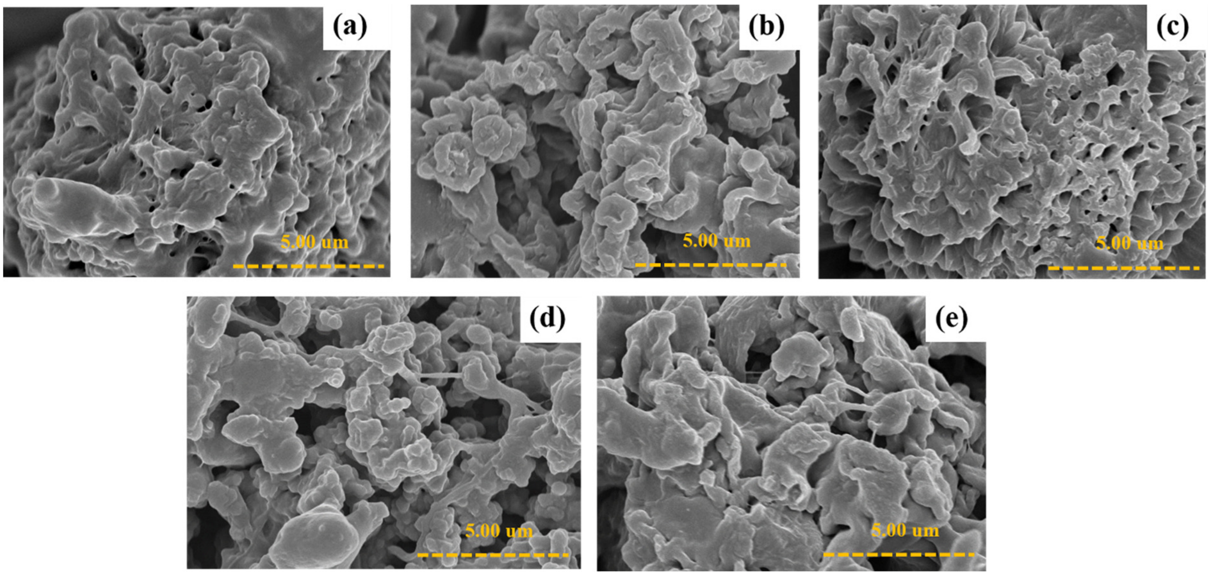

Figure S2. SEM images of P3HT synthesized by chemical oxidative polymerization with various PTSA concentrations: (a) P3HT_PTSA at 2 CMC; (b) P3HT_PTSA at 4 CMC; (c) P3HT_PTSA at 6 CMC; (d) P3HT_PTSA at 8 CMC; and (e) P3HT_PTSA at 10 CMC.

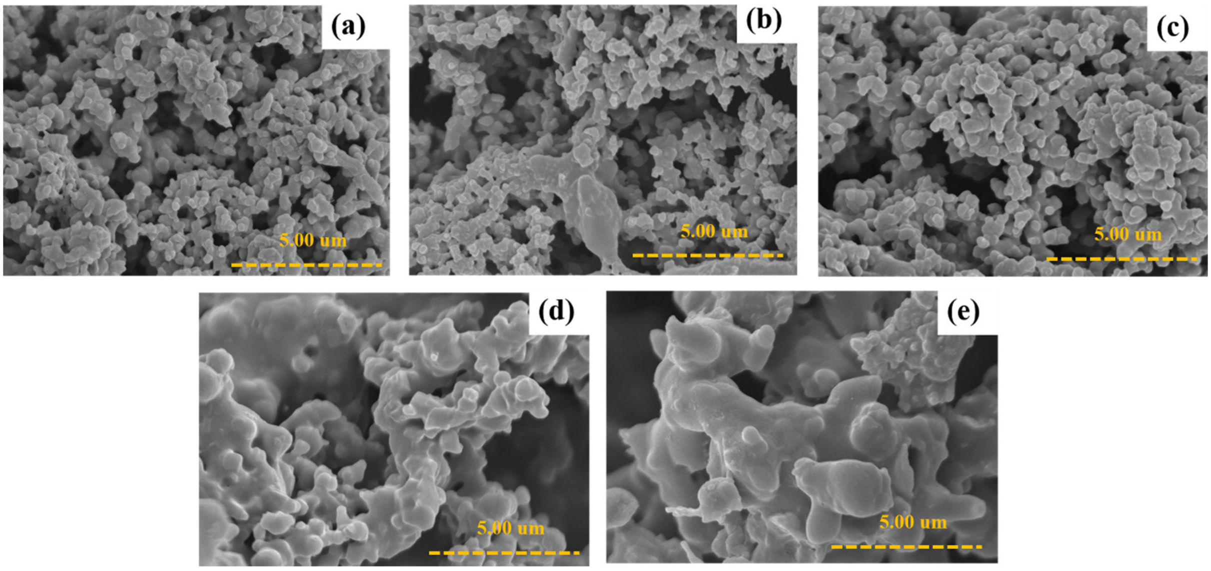

Figure S3. SEM images of P3HT synthesized by chemical oxidative polymerization with various SDS concentrations: (a) P3HT_SDS at 2 CMC; (b) P3HT_SDS at 4 CMC; (c) P3HT_SDS at 6 CMC; (d) P3HT_SDS at 8 CMC; and (e) P3HT_SDS at 10 CMC.

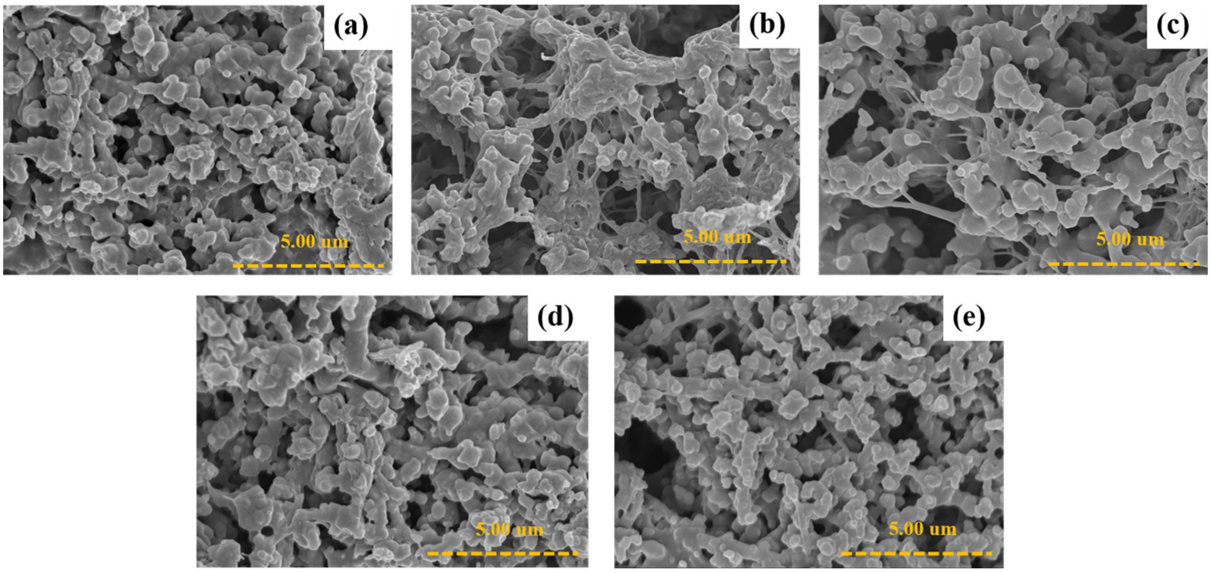

Figure S4. SEM images of P3HT synthesized by chemical oxidative polymerization with various AOT concentrations: (a) P3HT_AOT at 2 CMC; (b) P3HT_AOT at 4 CMC; (c) P3HT_AOT at 6 CMC; (d) P3HT_AOT at 8 CMC; and (e) P3HT_AOT at 10 CMC.

Table S1 XPS data of P3HT synthesized with and without surfactant at various oxidant :mole ratios at the polymerization time of 12 h

**Condition/Sample**	**Element (%At)**				**Cl/S**	**Doping level (%)**
	**C 1s**	**S 2p**	**O 1s**	**Cl**		
**Effect of oxidant at polymerization time 12 h**						
3HT :$FeCl_3$ mole ratio of 1 :2.5	82.57	3.06	14.13	0.24	0.0784	7.8
3HT :$FeCl_3$ mole ratio of 1 :3	82.61	6.30	9.45	1.64	0.2603	26.0
3HT :$FeCl_3$ mole ratio of 1 :3.5	89.33	5.64	4.52	0.50	0.0886	8.9
3HT :$FeCl_3$ mole ratio of 1 :4	81.27	3.03	15.48	0.22	0.0726	7.3
**Effect of surfactant types at 3HT :$FeCl_3$ mole ratio of 1 :3**						
P3HT-DBSA (6CMC)	86.18	9.14	2.71	1.97	0.2155	21.6
P3HT-PTSA (6CMC)	90.79	5.68	2.45	1.08	0.1901	19.0
P3HT-SDS (6CMC)	86.15	9.86	2.58	1.40	0.1419	14.2
P3HT-AOT (6CMC)	87.54	9.62	1.64	1.20	0.1247	12.5
**Effect of critical micelle concentrations at 3HT :$FeCl_3$ mole ratio of 1 :3 in DBSA**						
P3HT-DBSA (2CMC)	87.35	9.72	1.78	1.15	0.1183	11.8
P3HT-DBSA (4CMC)	85.58	8.59	4.25	1.58	0.1839	18.4

P3HT-DBSA (6CMC)	86.18	9.14	2.71	1.97	0.2155	21.6
P3HT-DBSA (8CMC)	90.25	6.29	2.48	0.99	0.1573	15.7
P3HT-DBSA (10CMC)	86.04	4.61	8.83	0.52	0.1127	11.3

Table S2 XPS data of P3HT synthesized at various doping levels

**Condition/Sample**	**Element (%At)**				**Cl/S**	**Doping level (%)**
	**C 1s**	**S 2p**	**O 1s**	**Cl**		
**De-doping/doping of Poly(3-hexylthiophene) by acid dopant ($HClO_4$)**						
deP3HT_DBSA	86.23	9.01	2.31	-	-	-
dP3HT_DBSA (1:1)	85.11	9.12	2.55	2.89	0.3168	31.7
dP3HT_DBSA (5:1)	84.21	8.12	2.46	3.45	0.4248	42.5
dP3HT_DBSA (10:1)	86.18	9.14	2.71	5.23	0.5722	57.2
dP3HT_DBSA (25:1)	85.21	8.99	2.03	4.25	0.4727	47.3
dP3HT_DBSA (50:1)	86.95	9.23	2.68	4.01	0.4344	43.4
dP3HT_DBSA (75:1)	86.72	9.20	2.11	3.38	0.3673	36.7

Table S3 Optical band gaps of the deP3HT, dP3HT_DBSA (1:1), dP3HT_DBSA (5:1), dP3HT_DBSA (10:1), dP3HT_DBSA (25:1), dP3HT_DBSA (50:1), and dP3HT_DBSA (75:1)

**Polymers (N_{HClO_4}/N_{3HT})**	**E_g (eV)**
deP3HT_DBSA (0:0)	5.95
dP3HT_DBSA (1:1)	1.36
dP3HT_DBSA (5:1)	1.20
dP3HT_DBSA (10:1)	1.10
dP3HT_DBSA (25:1)	1.26
dP3HT_DBSA (50:1)	1.40
dP3HT_DBSA (75:1)	1.48

The calculation of optical band gap energy

From the UV adsorption spectra, the band gap energy was calculated using the Tauc equation (1) (Sakunpongpitiporn et al., 2019):

$$\alpha h\nu = A(h\nu - E_g)^n \quad (1)$$

where α is the adsorption coefficient, hν is the photon energy (eV), h is the Plank's constant, *v* is the frequency (s^{-1}), A is a constant, E_g is the band gap energy (eV), and n is equal to 0.5 for the direct transitions. The adsorption coefficient (α) was calculated from the Beer-Lambert's relation (2):

$$\alpha = 2.303Ab/I \quad (2)$$

where Ab is the absorbance, and I is the sample path length. The extrapolation of the linear line from the plot between $(\alpha h\nu)^2$ on the y axis and hν on the x axis gives the E_g.

Reference

Sakunpongpitiporn, P.; Phasuksom, K.; Paradee, N.; Sirivat, A. Facile synthesis of highly conductive PEDOT: PSS via surfactant templates. *RSC Adv.* **2019**, *9*, 6363-6378.
